# Dr. Stephen Elmer Palmer

**Published:** 1917-03

**Authors:** 


**Dr. Stephen Elmer Palmer,** Univ. of Mich. 1872, died at his home in Elmira Feb. 9.

